# Mechanisms of Bacterial Superinfection Post-influenza: A Role for Unconventional T Cells

**DOI:** 10.3389/fimmu.2019.00336

**Published:** 2019-03-01

**Authors:** Christophe Paget, François Trottein

**Affiliations:** ^1^Centre d'Etude des Pathologies Respiratoires, Institut National de la Santé et de la Recherche Médicale U1100, Tours, France; ^2^Faculty of Medicine, Université de Tours, Tours, France; ^3^U1019-UMR 8204-CIIL-Centre d'Infection et d'Immunité de Lille, Université de Lille, Lille, France; ^4^Centre National de la Recherche Scientifique, UMR 8204, Lille, France; ^5^Institut National de la Santé et de la Recherche Médicale U1019, Lille, France; ^6^Centre Hospitalier, Universitaire de Lille, Lille, France; ^7^Institut Pasteur de Lille, Lille, France

**Keywords:** unconventional T cells, influenza A virus, secondary bacterial infection, *Streptococcus pneumoniae*, *Staphylococcus aureus*, immune suppression, barrier function, immunotherapy

## Abstract

Despite the widespread application of vaccination programs and antiviral drug treatments, influenza viruses are still among the most harmful human pathogens. Indeed, influenza results in significant seasonal and pandemic morbidity and mortality. Furthermore, severe bacterial infections can occur in the aftermath of influenza virus infection, and contribute substantially to the excess morbidity and mortality associated with influenza. Here, we review the main features of influenza viruses and current knowledge about the mechanical and immune mechanisms that underlie post-influenza secondary bacterial infections. We present the emerging literature describing the role of “innate-like” unconventional T cells in post-influenza bacterial superinfection. Unconventional T cell populations span the border between the innate and adaptive arms of the immune system, and are prevalent in mucosal tissues (including the airways). They mainly comprise Natural Killer T cells, mucosal-associated invariant T cells and γδ T cells. We provide an overview of the principal functions that these cells play in pulmonary barrier functions and immunity, highlighting their unique ability to sense environmental factors and promote protection against respiratory bacterial infections. We focus on two major opportunistic pathogens involved in superinfections, namely *Streptococcus pneumoniae* and *Staphylococcus aureus*. We discuss mechanisms through which influenza viruses alter the antibacterial activity of unconventional T cells. Lastly, we discuss recent fundamental advances and possible therapeutic approaches in which unconventional T cells would be targeted to prevent post-influenza bacterial superinfections.

## Influenza Virus Infection and Bacterial Superinfection

Respiratory infections are one the biggest health concerns worldwide. They account for a substantial rate of morbidity and mortality in Western and developing countries (1). Amongst respiratory pathogens, influenza viruses, commonly known as “the flu,” represent one the most important concern despite ongoing vaccine campaigns and anti-viral drugs. Each year, seasonal influenza infection affects 5 to 15% of the population and is a major contributor of pneumonia-related death worldwide (500,000 deaths per year) (2). Seasonal influenza is due to two main subtypes in humans, H1N1, and H3N2. Antigenic variations due to mutation in the hemagglutinin (HA) and neuraminidase (NA) genes, a phenomenon known as the antigenic drift, occur every year, and result in the circulation of new strains with sometime enhanced virulence and lethality potential. In parallel, in general every 10 to 20 years, new influenza subtypes distinct from circulating seasonal strains can emerge (due to antigenic shift) and provoke pandemic waves with sometime devastating consequences (3). Relative to seasonal influenza, pandemics exhibit a higher transmissibility and a higher rate of mortality, particularly among younger people who lack specific immunity against these new strains. Mortality attributed to influenza infection can be of high incidence during pandemics. During the Spanish flu (1918–1919), more than 40 million people died from influenza infection (4, 5). During the 2009 pandemic, influenza infection had a substantial impact on human mortality (3, 6). As discussed below, viral-bacteria pneumonia contribute significantly to morbidity and mortality during influenza epidemics and pandemics (5). Due to medical and economical burdens, and considering the threat of new pandemics and the emergence of antibiotic resistance, it is urgent to find novel options to fight against influenza infections and their complications, including secondary bacterial infections.

### Influenza a Virus: Main Characteristics

The influenza viruses (types A, B, and C) are negative sense single-stranded RNA viruses (7). These enveloped viruses belong to the family of *Orthomyxoviridae*. In humans, the most common cause of respiratory illness is influenza A virus (IAV) (8). To reach successful replication, sense messenger RNAs must be generated from the viral genome, a process due to the viral RNA polymerase. Sense messengers comprise eight RNA segments encoding eleven proteins (9, 10). The mature virion contains eight of these proteins surrounded by a protein envelope, which mainly includes two viral antigenic determinants: HA, which binds to terminal sialic acids expressed by airway and alveolar epithelial cells and NA, a critical enzyme necessary for releasing the viral progeny from infected cells. Eighteen HA subtypes and 11 NA subtypes have been identified to date. Two IAV subtypes (H1N1 and H3N2) along with one or two influenza B viruses co-circulate annually causing influenza epidemics. The primary targets (and site of replication) of IAV are airway and alveolar epithelial cells. Shortly after infection, the viral machinery, with the unintentionally help of host factors, is at work and generate the release of virions (11). Productive replication in epithelial cells results in cell death and to epithelial/endothelial damages leading to barrier rupture and exudation of fluids and proteins into the airways and alveolar spaces, greatly impairing gas exchanges (12). Meanwhile, an intense infiltration of immune cells (neutrophils, monocytes) occurs. Clinically, severe IAV infection can lead to acute respiratory distress syndrome, a severe form of respiratory failure associated with 40 % of mortality (5).

## Immune Response to IAV

Innate immune response is rapidly triggered after IAV infection (13–18). This relies on the presence of viral RNA in the cytosol of infected cells and on different and complementary innate sensors including Toll-like receptors (TLRs; primarily TLR3 and TLR7), retinoic acid inducible gene-1 and inflammasomes. Activation of these innate sensors results on the production of massive amounts of type I and type III interferons (IFNs) as well as interleukin (IL)-1β and IL-18. Type I and type III IFNs, though autocrine and paracrine (myeloid cells) effects, elicit the production of a myriad of IFN-stimulated genes that strongly participate in virus clearance. Meanwhile, activation of inflammasomes and NF- κB promotes the release of pro-inflammatory cytokines and chemokines and the subsequent recruitment and activation of numerous immune cells such as monocytes/macrophages and neutrophils. These events limit and/or prevent viral entry and replication and attenuate the severity of the disease. All of these responses however contribute to tissue injury. For instance, inflammatory monocytes greatly participate in epithelial cell damage and death (19, 20). On the other hand, although they participate in virus clearance, neutrophils are also strong contributors of lung damage and lethality (21). While epithelial cells are critical to initiate innate immunity, other resident sentinel cells also play a role in virus clearance. Alveolar macrophages promote the elimination of viruses through the phagocytosis of collectin-opsonised viral particles or infected apoptotic cells (efferocytosis) and production of inflammatory cytokines and chemokines (22, 23). In parallel, other resident and/or recruited innate immune cells, including natural killer cells, unconventional T cells, and innate lymphoid cells play a part in disease outcomes (mouse model of influenza), an effect associated—or not—with effector functions (24–33). On the other hand, depending on the infectious dose, some of them (e.g., NK cells) may also participate in immunopathology (34, 35). Several days after IAV entry and elicitation of innate immunity, a strong antigen (Ag)-specific CD8^+^ T cell response develops in the lungs. In this phenomenon, the migration of antigen-loaded CD103^+^ dendritic cells to the draining lymph nodes is critical. Even though Ag-specific CD8^+^ T cells are sufficient to contain viruses (e.g., through lysis of infected cells), they also contribute to alveolar epithelium and endothelium damage (36, 37).

## Resolution of Inflammation and Secondary Bacterial Infection

After the inflammatory burst and pulmonary tissue damage, a resolving/repair phase takes place, in general 7 to 14 days after the primary IAV infection. It leads to resolution of infiltrates and regeneration of damaged lung tissue thus restoring gas exchange. In this setting, murine studies of influenza infection suggested that CD8^+^ T cells, by producing the anti-inflammatory cytokine IL-10, are important to resolve inflammation (38). Activated macrophages can also promote the expansion of Foxp3-expressing regulatory T cells to suppress the deleterious production of inflammatory cytokines by neutrophils (39). By suppressing IL-17, a cytokine involved in neutrophil recruitment, type I IFNs also contribute to resolution of inflammation post-influenza. Recent data also indicate a role for M2 macrophages in this process (40). Unconventional T cells may also play a critical role in recovery from influenza infection (27, 33, 41, 42). Finally, through amphiregulin production, innate lymphoid cells restore airway epithelial integrity and tissue homeostasis during IAV infection (25). Resolution of inflammation during influenza infection is critical for lung resiliency and restoration of physiological functions (which can take several weeks). This regenerating response corresponds to a period of enhanced susceptibility to respiratory bacterial (particularly Gram-positive) infections. Indeed, this process creates a favorable environment to the emergence of opportunistic pathogens that can eventually result in bacterial superinfection, bacterial pneumonia and bacterial dissemination from the lungs. The two later are major contributors to lethality. Post-mortem examination of autopsy specimens collected during the last pandemic (as well as the 1918 pandemic) suggests that a substantial proportion of patients died from bacterial infections once the virus was cleared (5, 43). The most common bacteria found in autopsied individuals were *Streptococcus pneumoniae* (the pneumococcus) and *Staphylococcus aureus*, two major ubiquitous upper respiratory opportunistic pathogens. Two main mechanisms (mechanical and immunological) explain bacterial superinfection post-influenza: loss of the epithelial barrier function and altered innate immune defense. Before reviewing the role of “innate-like” unconventional T cells in this setting, we summarize the main mechanisms by which IAV favors secondary bacterial infection.

## Mechanical and Immunological Mechanisms Leading to Superinfection

Several excellent reviews have described the current mechanistic understanding of how IAV enhances susceptibility to secondary bacterial infections (44–49). Current data, mostly derived from experimental (mouse) models, point toward a multifactorial mechanism. Briefly, IAV disrupts the functions of the respiratory barrier by inducing, in a direct or indirect (through inflammatory monocytes) fashion, epithelial cell death, and by degrading mucins (20, 50). This alteration leads to exposure of new attachment sites for bacteria and allows bacterial translocation (51–53). Influenza A virus can also alter respiratory ciliary function, thereby impairing the clearance of aspirated bacteria from the lungs (54). As stated above, alteration of the innate immune response is critical in post-influenza bacterial superinfections. In particular, poor bacterial control in the context of prior IAV infection is due to the loss and/or dysfunction of macrophages and neutrophils (55–59). For these later, their ability to sense and clear (phagocytosis and killing activity) bacteria is profoundly altered (60–62). Along with these effector cells, dysfunction of natural killer cells also depresses host's antibacterial capabilities (63). Some cytokines are critical in bacterial superinfection. The immune-suppressive cytokine IL-10 inhibits the functions of macrophages and neutrophils (64, 65). IL-27, another immunosuppressive cytokine downstream of type I interferon receptor (IFNAR) signaling pathway also impairs innate immune response against secondary bacterial challenge (66, 67). Type I interferons, which are massively produced during IAV infection to limit viral replication, are also detrimental in bacterial superinfection. Mechanistically, they inhibit the production of chemokines (CXCL1 and CXCL2) important for the recruitment of macrophages and neutrophils to the lung and impair their phagocytic responses (57, 68, 69). Of note, type III IFNs (which share similarities with type I IFNs) favor bacterial superinfection post-influenza by disrupting the nasal microbiome, which often includes potential pathogens (70). The underlying mechanisms are still elusive but may depend on altered barrier functions of the nasal epithelium and dysfunctional innate defense. Although IFN-γ is critical in host defense against respiratory bacterial infections, it might favor secondary bacterial infection, for instance by decreasing the expression of the scavenger receptor MARCO on macrophages (56). In fact, the role of IFN-γ in bacterial superinfection is controversial since a protective role has also been suggested (71). Finally, IAV infection reduces, through signal transducer of activation and transcription-1 (STAT-1), the production of Th17-related cytokines, a critical family of cytokines involved in the control of respiratory bacterial infections (66, 72–75). Hence, the accumulating literature (experimental models) provides a clearer understanding of mechanisms leading to bacterial superinfection and suggests several targets to prevent it. In humans, impairment of innate immunity by pre-existing viral (IAV) infections has also been shown to hamper the control of carriage load and clearance of upper respiratory bacteria such as *S. pneumoniae* (76). This, along with mechanical defects (respiratory ciliary and barrier functions), may favor bacterial superinfection and secondary bacterial pneumonia. While some progresses have been made recently, much remains to be learned about the way that the virus alters pulmonary barrier functions and undermines protective antibacterial immunity during IAV-bacterial (co)infection. As outlined below, recent evidences suggest that unconventional T cell functions are targeted during IAV infection, a process that may be important in secondary bacterial infections.

## Unconventional T Lymphocytes

### Natural Killer T Cells

Natural killer T (NKT) cells represent a subset of lipid-reactive αβ T cells. In response to lipid Ags presented by the monomorphic Ag presenting molecule CD1d, NKT cells swiftly produce a large amount of cytokines, thus promoting and orientating immune responses (77). Lipid recognition by NKT cells is mediated by a conserved T cell receptor (TCR) repertoire. Natural killer T cells can be divided into two major populations: type I NKT cells and type II NKT cells. Type I NKT cells express a semi-invariant TCR α-chain (Vα14-Jα18 in mice and Vα24-Jα18 in humans) paired with a limited set of TCR β-chains (77, 78). These cells respond strongly to alpha-galactosylceramide (α-GalCer), a glycolipid under clinical development, particularly in cancer settings (79). Type I NKT cells also recognize endogenous lipids which are necessary for their selection in the thymus and for their activation at peripheral sites. Type I NKT cells can also react to microbial-derived lipids (80). Of importance, type I NKT cells also activate in response to a wide array of cytokines, including IL-12 and IL-23. Despite a relatively conserved TCR, type I NKT cells are heterogeneous and can be further divided into distinct subsets (81, 82). NKT cells produce a wide range of cytokines, with sometime opposite functions, a property that depends on the cell subset activated and on the nature of the stimulation (e.g., lipids and/or activating cytokines). Through this unique property, type I NKT cells can influence different types of immune responses ranging from T helper (Th)1-like, Th2-like, Th17-like, or T regulatory-like responses (83). This property is critical in pathological situations during which type I NKT cells can either exert positive or negative functions. Of note, type I NKT cells not only produce cytokines and display cytotoxic functions toward transformed cells and virally-infected cells (84). Type II NKT cells represent a much broader family of CD1d-restricted αβ T cells that react to lipids, but not to α-GalCer. They express a more diverse TCR repertoire that recognizes lipid Ags of various nature and origin (mammalian and microbial) (85). Due to the lack of specific tools, the functions of type II NKT cells have mainly been proposed indirectly by comparing the phenotypes observed in Jα18-deficient (which lack type I NKT cells) vs. CD1d-deficient (which lack both type I and type II NKT cells) mice in various settings. Type II NKT cells appear to share conserved phenotypic and functional features with type I NKT cells including an effector memory phenotype, cytotoxic potential and secretion of numerous cytokines/chemokines (85). Akin to type I NKT cells, type II NKT cells play important functions during (bacterial) infections. NKT cells, which are more abundant in mice relative to humans, populate both lymphoid tissues and mucosal sites, including the lungs (86, 87).

### Mucosal-Associated Invariant T cells

Mucosal-associated invariant T (MAIT) cells present many common features with NKT cells and γδ T cells including the capacity to rapidly react to non-peptide Ags. MAIT cells are defined by their restriction to the major histocompatibility complex class I-related molecule 1 (MR1) (88, 89). The majority of MAIT cells (referred to as classical MAIT cells) (90) express a semi-invariant TCR composed of a canonical TCRα-chain (Vα19-Jα33 in mice and Vα7.2-Jα33 in humans) associated with a restricted set of Vβ segments (88, 89, 91, 92). Through their TCR, MAIT cells recognize small intermediate metabolites from the riboflavin (vitamin B2) pathway of bacteria, mycobacteria and yeast (93–95). They can react to products derived from the non-enzymatic reaction between a riboflavin precursor and small aldehydes of both microbial and host origin. The high instability of these ligands has so far limited their use in the clinics. Reminiscent with NKT cells, MAIT cells can respond to TCR signals and/or to various activating cytokines, including IL-12 and IL-18 (96–98). Upon activation, MAIT cells produce large amounts of Th1- and Th17-related cytokines (99). Additionally, MAIT cells can kill bacteria-infected cells (100). Unlike NKT cells, MAIT cells are abundant in the blood (up to 10% of the T cell compartment) in humans. They are also present at mucosal sites, including the lungs (10% of respiratory mucosal T cells) (101), where they sense the environment and exert a role of sentinels of the immune system. Due to their scarce representation in common laboratory mouse strains (unlike NKT cells), understanding MAIT cell biology is challenging even using *Mr1*^−/−^ mice. To better assess their role in preclinical models, transgenic mice (*V*α*19i*Tg x Cα^−/−^) displaying high content of MAIT cells have been developed (102). Given their cytokine profile and cytotoxic potential, MAIT cells intuitively emerged as a specialized cell population in host defense against bacteria.

### γδ T Cells

γδ T cells represent approximately 1–10% of peripheral blood T cells in humans and are important components of both innate and adaptive immunity. They display vast effector and immune regulatory functions (103). Akin to other members of the unconventional T cell family, γδ T cells display a pre-activated status that allows rapid induction of effector functions following the detection of tissue stress (104–106). Another important feature of γδ T cells is their tropism for epithelial surfaces including lungs, to where they migrate shortly after development and persist as resident cells. They frequently express invariant or closely related γδ TCRs in a given tissue site (e.g., Vγ1, Vγ4, and Vγ6 in the mouse lung tissue), which confer them specific Ag recognition capabilities from one tissue to another (103). Reminiscent to other unconventional T cells, γδ T cells can kill infected cells and initiate adaptive immune responses through the release of substantial amounts of Th1- and Th17-related cytokines (103). Thus, γδ T cells have emerged as essential constituents of the antimicrobial immunity in both preclinical and clinical settings (107). While the Ags for mouse γδ TCRs have not been reported yet, human γδ T cell subsets (e.g., Vγ9Vδ2^+^ cells) can recognize both natural (of microbial and mammalian origins) and synthetic phosphoantigens (108). However, it is now clear that the phosphoantigens are not directly sensed by the γδ TCRs but rather require the involvement of butyrophilin BTN3A as an intermediate. The precise molecular mechanisms involved in this TCR-dependent γδ T cell activation are still a matter of debate (109). Whatever the mechanisms involved, phosphoantigens have been shown to strongly activate (*in vivo* and *ex vivo*) human Vγ9Vδ2 γδT cells to induce their proliferation and to increase their cytotoxic capacities as well as their cytokine secretion including IFN-γ and TNF-α. Given this, harnessing γδ T cell functions in therapeutic protocols is currently highly considered by clinicians especially in the context of cancer (110).

## Role of Unconventional T Cells in Respiratory Pneumococcal and Staphylococcal Infections

Evidence in both preclinical and clinical settings have suggested a key role for unconventional T cells in host response against lung bacterial pathogens. Here, we compared their mode of activation and functions during respiratory bacterial infections with a focus on the two major opportunistic pathogen bacteria implicated in bacterial superinfection post-influenza, namely *S. pneumoniae*, and *S. aureus*.

### Streptococcus pneumoniae

*Streptococcus pneumoniae* (also referred to as the pneumococcus) is the leading cause of community-acquired bacterial pneumonia worldwide (2 million deaths per year), with infants and the elderly exhibiting higher susceptibility. This Gram-positive bacterium, which comprises a group of more than 90 serotypes, colonizes asymptomatically nasopharynx of healthy individuals. However, when the immune equilibrium is broken, pneumococcus carriage can lead to mild disease such as otitis media or sinusitis and more occasionally turns into severe complications such as pneumonia, sepsis, and meningitis (111). *Streptococcus pneumoniae* is often found in biological fluids of hospitalized patients diagnosed for influenza infection as well as patients with exacerbated chronic lung inflammation (112, 113). Despite vaccination prevents pneumococcus spread and controls infections, the available vaccines have however some issues (114). In addition, the emergence of antibiotic-resistant strains represents an important threat for the management of pneumococcal infections in clinics (115).

**Figure 1:**
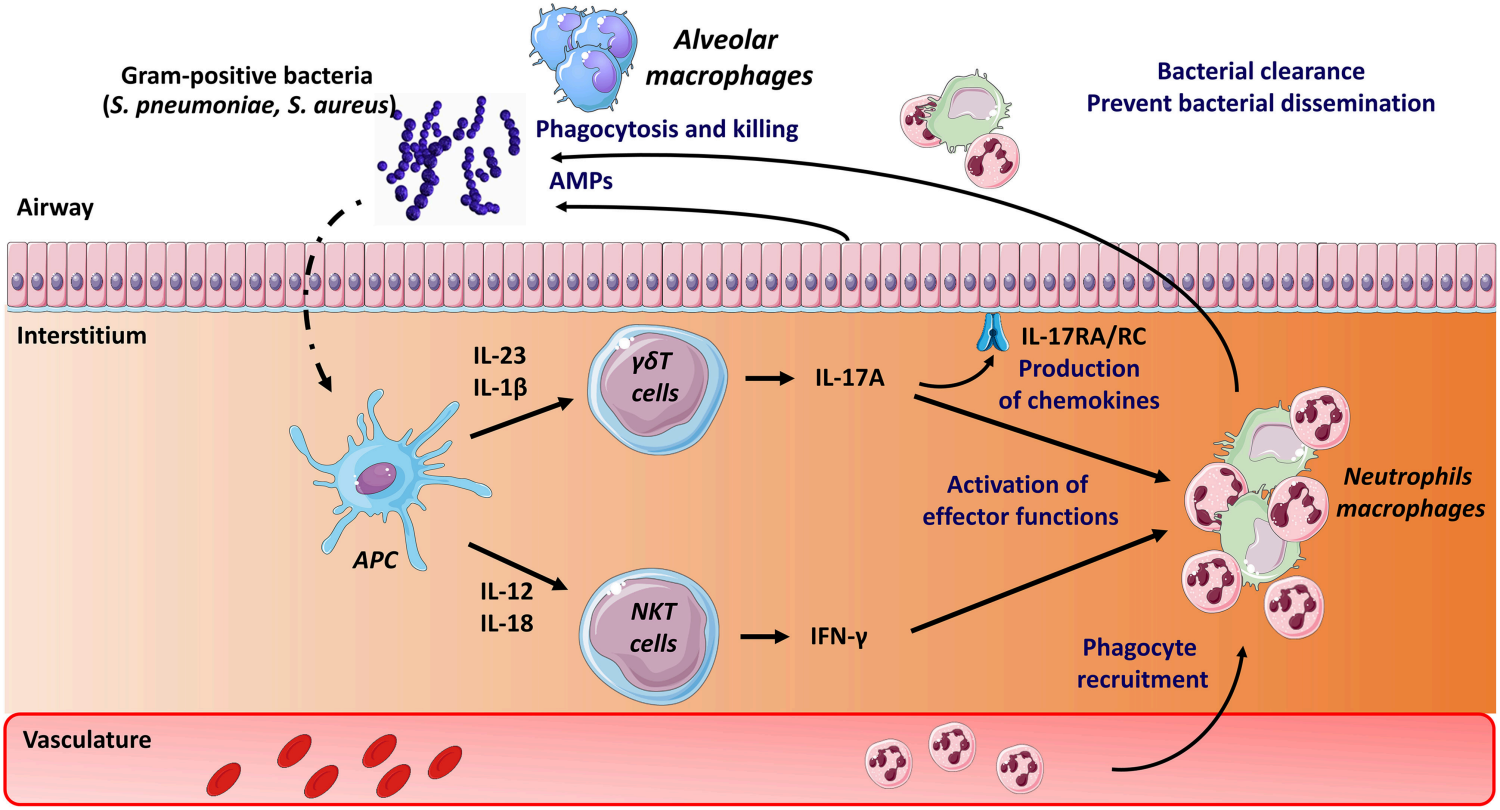
Mode of activation and role of unconventional T cells during pneumococcal and staphylococcal infections. Early after *S. pneumoniae* and *S. Aureus* infection, Ag presenting cells, including dendritic cells, produce a wide array of cytokines that activate pulmonary γδ T cells (IL-17A) and type I NKT cells (IFN-γ). Lipids from *S. pneumoniae* can also directly activate type I NKT cells through their TCR. The protective role mediated by γδ T cells and type I NKT cells comprises activation of innate effector cells, such as macrophages and neutrophils (IFN-γ, IL-17). The latter are rapidly recruited in response to chemokines produced, amongst other cell types, by epithelial cells (i.e., IL-17 receptor signaling pathways). Mechanisms leading to bacterial clearance include killing (bactericidal) activity of macrophages and neutrophils and release of antimicrobial peptides (AMPs). The potential role of MAIT cells in pneumococcal and staphylococcal infections is still unknown.

In the mouse system, both type I NKT cells and γδT cells activate in response to *S. pneumoniae* (Figure 1). While type I NKT cells produce IFN-γ early after pneumococcal challenge, γδT cells produce IL-17A (66, 74, 116–118). Activation of type I NKT cells during *S. pneumoniae* infection depends on pneumococcal-derived lipid(s) (α-glucosyldiacylglycerol), cytokines (IL-12) or both according to the strain studied (86, 116, 119). Of note, we and others have highlighted the role of CD103^+^ dendritic cells in the activation of type I NKT cells during pneumococcal infection (119, 120). The mechanisms through which murine γδT cells activate (IL-17) mainly depends on IL-1β and IL-23 (117, 121). The lack of type I NKT cells (*J*α*18*^−/−^ mice) (119, 122, 123) or γδT cells (*Tcrd*^−/−^ mice) (66, 117) results in higher bacterial loads and mortality. The underlying mechanisms of this protective activity rely on IFN-γ and IL-17 secretion and on the early recruitment of neutrophils. Hence, both type I NKT cells and γδT cells play a natural positive role in host defense against experimental pneumococcal infection (Figure 1). The potential role of type II NKT cells and MAIT cells during pneumococcal infection is still elusive. Of interest, *S. pneumoniae* expresses enzymes involved in the synthesis of riboflavin metabolites (124, 125) and human MAIT cells produce, in an MR1-dependent manner, IFN-γ in response to dendritic cells and airway epithelial cells exposed to *S. pneumoniae* (126, 127). Using Vα19iTg x Cα^−/−^ mice, a small proportion of lung MAIT cells were shown to produce IFN-γ and IL-17A during pneumococcal infection (127). Although a more detailed kinetic analysis is required, these levels were relatively low compared to those produced by NKT cells and γδT cells. The use of *Mr1*^−/−^ or Vα19iTg x Cα^−/−^*Mr1*^−/−^ mice will be instrumental to address the role of MAIT cells during experimental pneumococcal infection.

The potential effects of exogenous activation of unconventional T lymphocytes on pneumococcal infection have been examined. Inoculation of the type I NKT cell superagonist α-GalCer protects against lethal pneumococcal infection in the mouse system (123, 128). Mechanistically, this protective activity relies on respiratory CD103^+^ dendritic cells and on both IFN-γ and IL-17A production and neutrophils (128). The potential effect of exogenous γδ T cell and MAIT cell activation on host defense against pneumococcal infection is presently unknown. Despite emerging evidence for a critical role in host response to pneumococcus in experimental models, information regarding the phenotype and dynamics of unconventional T cells in patients with severe *S. pneumoniae*-driven pneumonia are rather limited. Of note, the level of circulating MAIT cells in critically ill patients with severe bacterial infection is markedly decreased compared to age-matched healthy controls (129). Although this decrease is less striking in patients with streptococcal infections, these data suggest that MAIT cells may migrate into the lungs, and thus may exert a potential role during pneumococcal infection.

### Staphylococcus aureus

*Staphylococcus aureus* is a Gram-positive bacterium with a potent pathogenic potential to cause a variety of community and hospital-acquired infections. In normal conditions, it commonly colonizes the upper airways. Under certain circumstances, including influenza infection, it can cause localized and serious invasive infections, as well as a severe septic shock syndrome (130). The frequency of these infections is increasing. The ability of *S. aureus* to form biofilms and the emergence of multidrug-resistant strains (e.g., methicillin-resistant *Staphylococcus aureus*) are the main reasons why their treatment is becoming more difficult. The capacity of *S. aureus* to become pathogenic is related to the expression of virulence factors, among which the production of a wide variety of toxins. Staphylococcal superantigens (SAgs) constitute a family of potent exotoxins secreted by *S. aureus* (131). They can cross-link MHC class II molecules with TCRs to stimulate an uncontrolled polyclonal activation of T lymphocytes (cytokine storm), potentially leading to severe illnesses including toxic shock syndrome.

Despite a relatively poor literature in the field, unconventional T cells might play role during *S. aureus* infection. They also recently emerged as potential targets of Staphylococcal SAgs. Indirect evidence suggest that IL-17 production by γδ T cells might be important in the control of *S. aureus* lung infection (72, 73) (Figure 1). Mice lacking γδ T cells have a reduced ability to clear bacteria and to control pulmonary inflammation (132). The role of NKT cells and MAIT cells in the control of *S. aureus* is still unknown. On the other hand, emerging evidence suggest that unconventional T cells (at least NKT cells and MAIT cells) are involved in toxic shock syndrome induced by Staphylococcal SAgs. Intranasal inoculation of Staphylococcal enterotoxin B promotes the activation of type I NKT cells and lung injury (133). Staphylococcal enterotoxin B activates mouse and human type I NKT cells via a MHC class II (but not CD1d) Vβ8-dependent pathway (134). More recently, Szabo et al., using SAg-sensitive HLA-DR4-transgenic mouse demonstrated that type I NKT cells are pathogenic (toxic shock syndrome) in response to Staphylococcal enterotoxin B (135). Of interest, administration of a Th2-polarizing glycolipid agonist for type I NKT cells reduced morbidity and mortality. Type I NKT cells may therefore constitute an attractive therapeutic target in SAg-mediated illnesses. Mouse and human MAIT cells can also activate in response to Sags in a largely TCR-independent, cytokine-driven manner (136). They produce a huge amount of pro-inflammatory cytokines and thereafter become unresponsive to stimulation with bacterial Ags. Through this mechanism, they might participate in cytokine storm and subsequent immunosuppression. Akin to type I NKT cells, MAIT cells may therefore provide an attractive therapeutic target for the management of both early and late phases of severe SAg-mediated illnesses.

## Role of Unconventional T Cells in Bacterial Superinfection Post -Influenza

As outlined below, mouse models of viral-bacterial infection have been used to assess the role of unconventional T cells in bacterial superinfection post-influenza. These cells are activated during influenza infection (24, 27–33, 137) and, through their ability to control barrier function, they may limit bacterial superinfection. On the other hand, although activation during influenza infection may preset their antibacterial effector functions, immune suppression arising from influenza counteracts their antibacterial potentials.

### Role of Unconventional T Cells in Pulmonary Barrier Functions

Disruption of the pulmonary barrier functions strongly contributes to enhanced bacterial colonization, bacterial superinfection and bacterial pneumonia in the context of prior influenza. Emerging evidences suggest that unconventional T cells play a natural role in the maintenance of tissue integrity and/or in tissue repair processes (138). Recent studies have addressed the role of unconventional T cells in tissue homeostasis and barrier functions during experimental influenza. Type I NKT cells and γδ T cells produce the tissue protective cytokine IL-22 (through IL-1β- and IL-23) during the early course of IAV infection (24, 139). Although IL-22 does not affect viral loads during influenza, several independent groups have demonstrated the protective effect of IL-22 against epithelial damages caused by viral replication (24, 139–143). The mechanisms through which IL-22 prevents epithelial barrier dysfunction during influenza infection might include an inhibitory effect on the recruitment of inflammatory monocytes and a direct effect on the expression of genes involved in barrier functions (143). Interleukin-22 might also participate in airway epithelial regeneration and barrier repair (141, 142). Interestingly, through its protective effect on barrier functions, IL-22 reduces secondary bacterial infection (139, 143). Of note, MAIT cells have recently been reported to accumulate in the lungs and to activate (through IL-18) during experimental IAV infection, a process associated with protection against a lethal viral challenge (33). Although not firmly established, MAIT cell activation during IAV infection may reduce pulmonary epithelial damage and reinforce barrier functions. Hence, by rapidly producing tissue protective factors, unconventional T cells, including NKT cells, γδ T cells, and MAIT cells, may limit the extent of secondary bacterial infection post-influenza. It is noteworthy that NKT cells can also indirectly activate the synthesis of protective barrier factors by other cells (e.g., amphiregulin by group 2 innate lymphoid cells) (25, 41). These functions might be exploited fortherapeutic purposes.

### Role of Unconventional T Cells in Pulmonary Innate Responses

**Figure 2:**
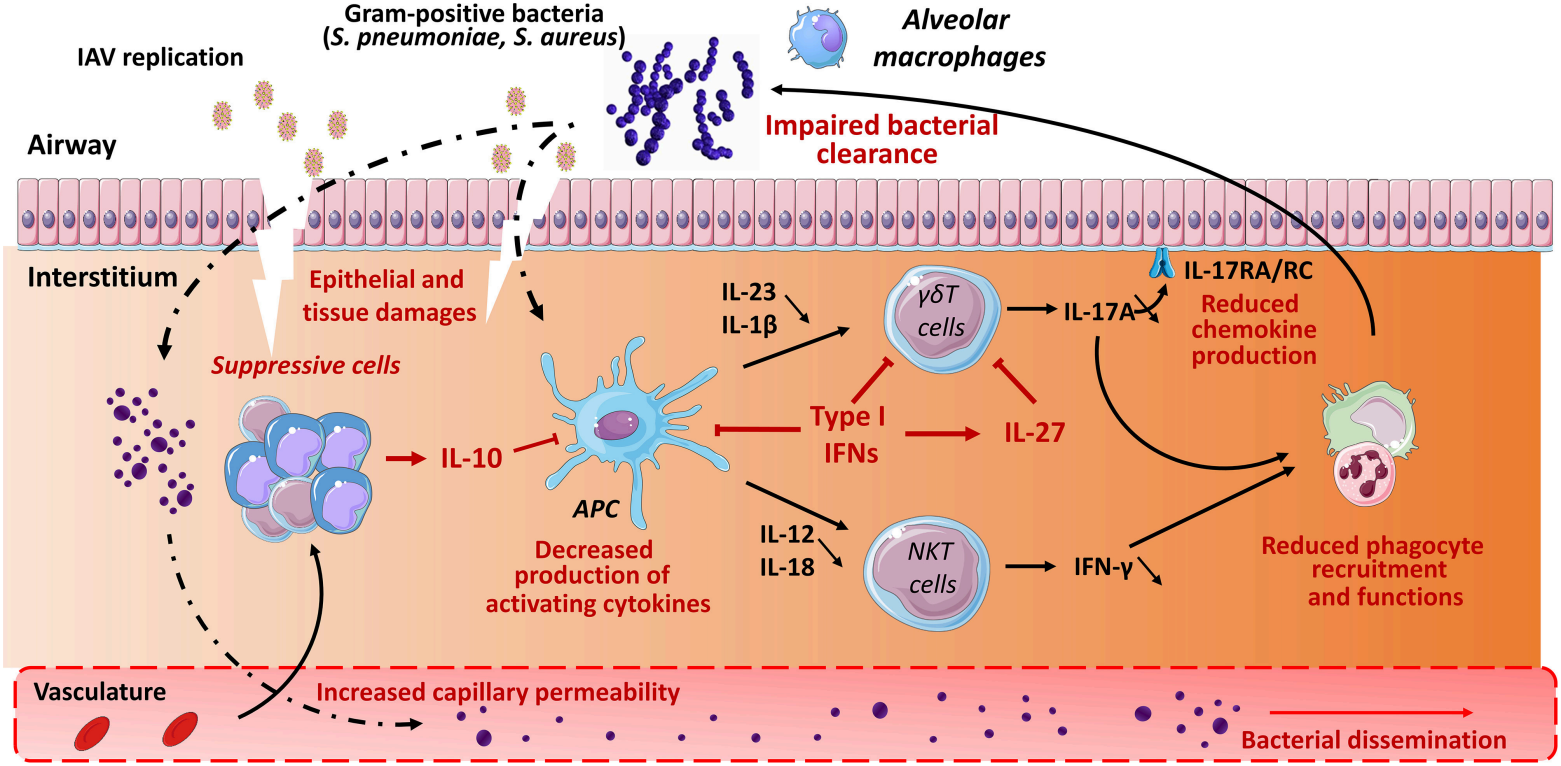
Role of unconventional T cells in bacterial superinfection post-influenza. Influenza A virus replicates in epithelial cells, thus leading to cellular damage and pulmonary inflammation. Along with typical inflammatory cells (neutrophils, inflammatory monocytes), the lungs is infiltrated with various populations of immune suppressive cells expressing for instance IL-10. The later inhibits the production of IFN-γ by type I NKT cells. Meanwhile, due to reduced IL-1β and IL-23 production, γδ T cells display a defective ability to release IL-17A. Multiple mechanisms are involved including a direct role for type I IFNs on Ag presenting cells (reduced IL-1β and IL-23) and γδ T cells (reduced IL-17A) and a promoting effect of type I IFNs on IL-27 synthesis. Interleukin-27 in turn targets γδ T cells to reduce IL-17A production. During IAV infection, there is also a numeric and/or functional defect of alveolar macrophages and neutrophils. As a result, the development of respiratory bacteria in the lung compartment is not controlled, leading to severe bacterial pneumonia and bacterial dissemination from the lungs. The potential positive role of γδ T cells and type I NKT cells (IL-22) in the maintenance of the epithelial barrier is not mentioned. The potential role of MAIT cells in bacterial superinfection is still unknown.

Alteration of innate immune defense also strongly contributes to bacterial superinfection post-influenza. As stated above, unconventional T cells play a part in host defense against *S. pneumoniae* and *S. aureus*. Here, we summarize host factors that (may) compromise their protective functions in the context of double viral and bacterial infection (mouse system). In this setting, IL-10 and type I IFNs appear to play a relevant role. During IAV infection, IL-10 is massively produced by innate and adaptive immune cells. This includes CD4^+^ (including regulatory T cells) and CD8^+^ T cells as well as NK cells and myeloid cells, mostly inflammatory monocytes (38, 119). Our data indicate that in the context of prior influenza, type I NKT cells fail to produce the protective cytokine IFN-γ (Figure 2), an effect associated with worse secondary pneumococcal infection (119). Blockade of IL-10 rescues activation of type I NKT cells (through restoration of IL-12 production by Ag-presenting cells), reduces bacterial outgrowth and dissemination and improves disease outcomes. Hence, the lack of type I NKT cell activation participates, at least in part, to bacterial (pneumococcal) superinfection post-influenza. Along with IL-10, type I IFNs favor bacterial superinfection post-influenza (57, 66, 68, 72–74, 144). γδ T cells appear to be the main target of type I IFNs. In the context of double IAV-bacterial (both pneumococcal and staphylococcal) infection, γδ T cells fail to secrete IL-17 in a type I IFN-dependent manner (66, 72, 74) (Figure 2). This ultimately leads to altered neutrophil recruitment and activity and to inhibition of the IL-17 antimicrobial pathway, including production of antimicrobial peptides. In this setting, the mode of action of type I IFNs is multiple. Type I IFNs can block the secretion of Th17-promoting cytokines IL-1β and IL-23 by Ag-presenting cells (72, 73). On the other hand, type I IFNs can directly target γδ T cells, via IFNAR, to inhibit IL-17 production (74) (Figure 2). Finally, type I IFNs indirectly inhibit IL-17 release by γδ T cells by promoting IL-27 production (66, 67). IL-27 targets γδ T cells to decrease expression of the IL-17-promoting factors RORγt and IL-23 receptor (66). The later mechanism is probably dominant as exogenous administration of IL-27 reverses the resistance phenotype of IFNAR-deficient mice upon post-influenza bacterial infection via down-regulating IL-17 production by γδ T cells and neutrophil response. Whether MAIT cell functions are affected by influenza, for instance through IL-10 or type I IFNs, is still ignored. Type III IFNs have also been shown to favor bacterial superinfection post-influenza (70). Regarding the role of unconventional T cells in barrier functions and innate antibacterial immunity, one can speculate that type III IFNs (like type I IFNs) also alter the functions of these cells to favor bacterial superinfection.

### Human Studies

Whilst the use of experimental models suggests a role for NKT cells and γδ T cells in bacterial superinfection post-influenza (the potential role of MAIT cells has not yet been appreciated), few studies have so far investigated unconventional T cells during human influenza and secondary infections. MAIT cells are more abundant in the blood relative to NKT cells and, to a lesser extent, γδ T cells. Compared to healthy donors, the frequency of circulating MAIT cells decreased in patients hospitalized for severe pneumonia due to infection with the Asian lineage avian IAV (H7N9) (96). Of interest, individuals who recovered from pneumonia had a higher level of circulating MAIT cells compared with patients who succumbed (96). This study suggested a protective role of MAIT cells in human influenza. Another clinical study confirmed the reduced peripheral blood MAIT cell frequencies (and enhanced granzyme B expression) in patients with acute IAV infection (2009 H1N1 pandemic) (145). This decrease was even more pronounced in critically ill patients admitted in intensive care unit compared to patients with mild symptoms. Reduction of MAIT cell numbers during acute human influenza infection (critically ill patients) could impair protective anti-bacterial immunity increasing the risk of secondary bacterial infections, which would enhance disease severity and mortality. The frequency, number and functional state of NKT cells and γδ T cells during human influenza have not yet been examined. In influenza vaccinated individuals, γδ T cells proliferate and activate although this intensity weakens with age (146).

## Therapeutic Opportunities

The unique biologic features of unconventional T cells are now being harnessed in the fight against cancer (79, 110, 147). Although this field is still in its infancy, exploitation of these cells in the management of lung infections appears to have therapeutic promise (148). Targeting unconventional T cells has several advantages. Firstly, the cells' restriction to non-polymorphic Ag-presenting molecules renders most patients eligible for unconventional T cell-based therapy using universal ligands. The question of whether unconventional T cells are potential immune targets in post-influenza bacterial superinfections has recently been addressed in preclinical models. The results suggest that these cells can indeed be exploited therapeutically. One major obstacle is the difficulty in balancing the induction of effective bacterial clearance and the avoidance of excessive inflammation. Cytokine-based strategies, neutralizing antibodies, and treatment with agonists that are specific for unconventional T cells have generated promising results over the last few years (mouse system).

The overexpression and/or inoculation of IL-23 or IL-1β restores the defective production of IL-17 by γδ T cells and T helper cells, and improves the clearance of pneumococci and staphylococci (72, 73). Furthermore, neutralization of IL-10 and IL-27 by blocking antibodies during the course of influenza restores the respective abilities of NKT cells and γδ T cells to combat secondary bacterial infections (66, 119). It is important to note that this approach is associated with better disease outcomes, including higher survival rates. In the mouse system, it has been suggested that treatment with the superagonist α-GalCer can enhance the beneficial activity of type I NKT cells. Inoculation of α-GalCer during IAV infection markedly reduces the bacterial (pneumococcal) burden in the lungs and bacterial dissemination from the lungs (149). However, the efficacy of this type of treatment is limited by its narrow therapeutic window; on day 7 (when susceptibility to superinfection peaks), α-GalCer has no effect. This is due to the disappearance of CD103^+^ dendritic cells (150)–a critical population involved in activation of type I NKT cells in the lungs—at this time point (128). In contrast, α-GalCer treatment early in the IAV infection (on day 4) or during the resolution phase (day 14) is associated with lower pneumococcal outgrowth and dissemination. Less intense viral-bacterial pneumonia and a lower morbidity rate were observed in superinfected mice treated with both α-GalCer and the anti-inflammatory corticosteroid dexamethasone (149). However, this combination therapy was not associated with a lower mortality rate during secondary bacterial superinfection. In contrast to type I NKT cells, the potential effects of agonists on γδ T cells and MAIT cells in the context of post-influenza secondary bacterial infections have yet to be investigated.

Although the above-mentioned findings (the restoration of NKT cell and/or γδ T cell functions) have revealed a novel aspect of immunotherapy against superinfection in animal models, their clinical relevance remains to be proven. In the search for an effective balance between effective bacterial clearance and the avoidance of excessive inflammation, it is likely that additional therapeutic approaches (e.g., anti-inflammatory drugs) will have to be implemented. One can also speculate that combination treatment with antibiotics might enhance the efficacy of immunotherapy. It was recently shown that the application of a combination of antibiotics and immune stimulators (e.g., Toll-like receptor agonists) improved the outcome of post-influenza bacterial superinfection in a murine system (151). On the same lines, it would be useful to study the effects of a combination of an agonist (e.g., α-GalCer) and an antibiotic. Another key challenge relates to cell targeting. As discussed above, unconventional T cells are heterogeneous, and comprise subpopulations with sometime opposite functions. It will be necessary to target subpopulations of interest (e.g., IL-17 producers) more accurately by engineering T-helper-polarizing agonists (which have only been developed for NKT cells so far) and/or co-factors polarizing their functions. This would open the way to immunotherapies tailored to match a patient's immune profile. Importantly, it seems that this type of treatment must also take account of the nature of secondary bacterial infection. As discussed above, Staphylococcal SAgs have a critical role in the pathogenesis of *S. aureus* infections. In this setting, antagonists or T-helper-polarizing agonists could be used to manipulate type I NKT cells and MAIT cells—both of which are hyper-responsive to SAgs.

## Conclusions and Perspectives

Unconventional T cells have attracted growing interest from researchers and clinicians. The literature on the cells' roles in immune and inflammatory responses has grown tremendously over the last 10 years. In view of their immunoregulatory potential, unconventional T cells are well poised to help fight lung infections and the latter's complications. However, there is a paucity of preclinical and clinical research on the cells' potential roles in the context of influenza and secondary bacterial infections. Further research into (i) the role of unconventional T cells in bacterial (super) infections of the respiratory tract and (ii) how influenza modulates the cells' functions is now needed. Furthermore, the use of novel mouse models will be essential for defining the respective roles of unconventional T cells and their subsets in influenza and secondary bacterial infections. Given that the mechanisms of post-influenza bacterial superinfections are multifactorial (with the exploitation of mechanical and/or immune alterations in the host), future therapeutics will probably have to include several components that target several host factors in addition to the viruses and bacteria themselves. Although this approach is in its infancy, the manipulation of unconventional T cells during influenza (cytokines, α-GalCer) has shown its potential in the fight against secondary infections. As mentioned above, this strategy is not problem-free, and must be considered with caution. Research on the effects of combining immunostimulatory factors with antimicrobial drugs (e.g., antibiotics) should be encouraged, and might help to lessen the development of drug resistance. Given the physiological role of unconventional T cells in tissue repair and barrier functions, strategies for promoting these functions might also be of value. Lastly, given the role of type I NKT cells and MAIT cells in the cytokine storm that follows exposure to Staphylococcal SAgs, the manipulation of these cells might help to control the outcomes of secondary staphylococcal infection—including necrotizing pneumonia. As discussed in this review, there is also a critical knowledge gap between preclinical and clinical studies; hence, analyses of the frequency/number and functional states of patients' unconventional T cells should be encouraged. Counts of circulating unconventional T cells are not negligible; considering the critical role they exert in many diseases, one can expect to see some major breakthroughs in the near future. Promising research initiatives might include a complete analysis of the whole family of unconventional T cells, i.e., NKT cells, group 1 CD1-restricted T cells, MAIT cells and γδ T cells. High-throughput RNA sequencing (at the bulk population and single-cell levels) and the computer modeling of cytokine signatures in patients should also be encouraged. Although the work will be time-consuming and arduous, it might translate into improved clinical outcomes.

In conclusion, we critically analyzed the available evidence on the potential role of unconventional T cells in post-influenza bacterial superinfections. In view of these cells' extraordinary immunostimulatory and immunoregulatory properties and the proven safety of unconventional T cell agonists, further research in this field should be encouraged.

## Author Contributions

All authors listed have made a substantial, direct and intellectual contribution to the work, and approved it for publication.

### Conflict of Interest Statement

The authors declare that the research was conducted in the absence of any commercial or financial relationships that could be construed as a potential conflict of interest.
